# Recent Advances in the Synthesis of Ibuprofen and Naproxen

**DOI:** 10.3390/molecules26164792

**Published:** 2021-08-07

**Authors:** Min-Woo Ha, Seung-Mann Paek

**Affiliations:** 1 Jeju Research Institute of Pharmaceutical Sciences, College of Pharmacy, Jeju National University, 102 Jejudaehak-ro, Jeju 63243, Jeju-do, Korea; minuha@jejunu.ac.kr; 2 Interdisciplinary Graduate Program in Advanced Convergence Technology & Science, Jeju National University, 102 Jejudaehak-ro, Jeju 63243, Jeju-do, Korea; 3 College of Pharmacy and Research Institute of Pharmaceutical Sciences, Gyeongsang National University, 501 Jinju-daero, Jinju 52828, Gyeongnam-do, Korea

**Keywords:** NSAIDs, ibuprofen, naproxen, synthesis

## Abstract

Herein, we review the recent progress in the synthesis of representative nonsteroidal anti-inflammatory drugs (NSAIDs), ibuprofen and naproxen. Although these drugs were discovered over 50 years ago, novel practical and asymmetric approaches are still being developed for their synthesis. In addition, this endeavor has enabled access to more potent and selective derivatives from the key frameworks of ibuprofen and naproxen. The development of a synthetic route to ibuprofen and naproxen over the last 10 years is summarized, including developing methodologies, finding novel synthetic routes, and applying continuous-flow chemistry.

## 1. Introduction

Inflammation is a natural defense response of the immune system to non-self-recognition or unusual self-abnormality [1]. Although this crucial process protects our body from unusual disorders, it sometimes causes unwanted physical symptoms such as pain and heat. In this regard, a therapeutic method for blocking uncontrolled inflammation has been studied [2]. Steroids are the most powerful anti-inflammatory medicines used for this purpose [3]. However, steroidal therapy for anti-inflammation causes additional side effects such as anabolism, sexual dysfunction, and problematic mineral absorption [4,5]. Since steroids are sensitive to hormones in various organs, non-steroidal therapy has also been pursued. Acetylsalicylic acid (ASA), known as aspirin, has been used for this purpose, although its exact mechanism was only uncovered almost 70 years after its first discovery in 1897 [6].

**Figure 1:**
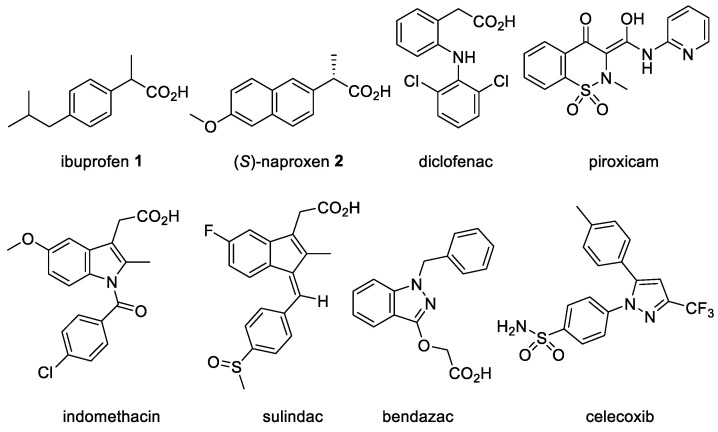
Structure of representative NSAIDs.

ASA exerts its effects through the formation of a covalent bond between an acetyl group in ASA and a serine residue in the active site of the cyclooxygenase enzyme (COX). This irreversible bond formation inhibits the activity of COX in the production of prostaglandin, which is pivotal in inflammation processes [7]. However, this irreversible inhibition of target proteins causes severe side effects such as gastrointestinal ulceration [8,9] and bleeding [10]. Since COX2 was reported in 1991 [11], numerous studies have been performed to realize more selective and potent medicines. Currently, many medicines are available in this class. These are nonsteroidal anti-inflammatory drugs (NSAIDs) as shown in Figure 1 [12,13].

As NSAIDs prevail as pain relievers, they have become highly popular drugs in the pharmaceutical market. Their large market has forced the pharmaceutical community to discover advanced synthesis routes for NSAID skeletons [14,15,16]. From a structural viewpoint, NSAIDs skeletons are usually classified as being an aromatic acetic acid or propionic acid skeleton that contains an additional chiral center. Among the aromatic propionic acid skeletons, ibuprofen and naproxen are the most widely used. Ibuprofen was developed in the 1960s [17] and was valued at around 300 million US dollars in 2020 [18]. In addition, it was prescribed more than 2.4 million times in the USA in 2018 [19] and was the most prescribed NSAID in that year. The second most prescribed NSAID was naproxen, which was developed in the 1970s. Considering that both ibuprofen and naproxen can be purchased without a prescription, it is believed that their medicinal benefits have significantly improved patients’ lives.

Ibuprofen and naproxen have an aryl-propanoic acid skeleton, possessing stereogenic center. (Figure 1) Although it features a relatively small structure, its synthesis has been studied to reduce synthetic steps, improve reaction conditions, increase reaction scales, and acquire facial selectivity. It is also interesting that they were sometimes prepared for the purpose of demonstration of synthetic methodologies, which were recently developed.

In this review, we present novel endeavors to improve the synthesis of ibuprofen or naproxen over the last 10 years. Although synthetic procedures have been well established, more advanced synthesis routes are still required to find efficient preparation methods and potent derivatives of ibuprofen and naproxen [20].

## 2. Classical Synthesis of Ibuprofen

**Scheme 1:**
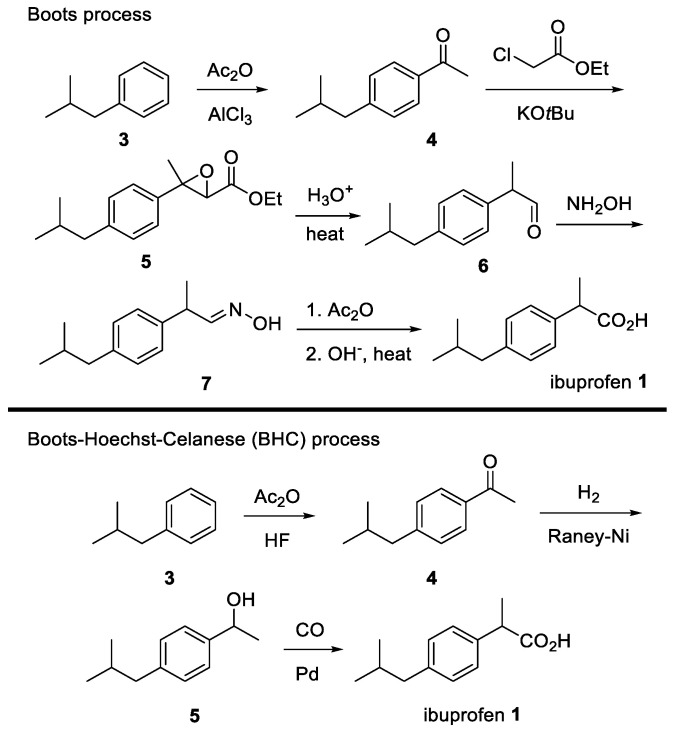
Classical synthesis of ibuprofen.

The classical synthesis of ibuprofen is shown in Scheme 1. When the Boots Pure Drug Company developed ibuprofen in 1961, it was prepared in six steps, including the use of toxic aluminum chloride in the early stage [21]. However, in 1992, the Hoechst Company protocol improved this procedure by using recyclable hydrogen fluoride as an alternative to aluminum chloride. Moreover, the synthesis was accomplished using a simple carbon monoxide (CO) insertion method without additional hydrolysis or dehydration [22]. With a three-step procedure, ibuprofen can be supplied worldwide. However, there is still an unmet need for a simpler, more efficient, and stereospecific route.

## 3. Recent Synthetic Advances in Ibuprofen

**Scheme 2:**
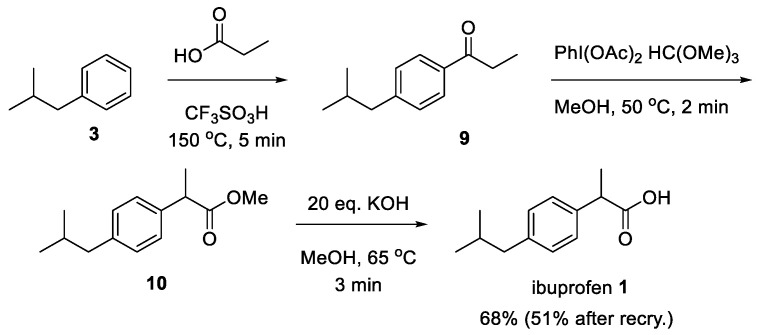
Synthetic strategy for continuous-flow synthesis of ibuprofen.

The application of continuous-flow synthesis for ibuprofen is shown in Scheme 2 [23]. This synthetic route adapts the iodine-mediated 1,2-aryl migration reaction. McQuade group used trifluorosulfonic acid as a reaction catalyst instead of the conventional reagent, aluminum chloride, to achieve continuous-flow synthesis. Aluminum byproducts are incompatible in further steps. The next step was the 1,2-aryl migration reaction. After the model study and careful survey of reaction conditions, substrate ketone **9** could be quantitatively converted into methyl ester **10**. Finally, saponification of the methyl ester functionality afforded ibuprofen as a light orange solid. It is noteworthy that the reaction could be completed in 10 min using a flow reactor with a 68% overall yield (51% after recrystallization). Moreover, the simplicity and efficiency of this synthetic process makes it likely to satisfy the unmet need for improved ibuprofen synthesis [24].

**Scheme 3:**
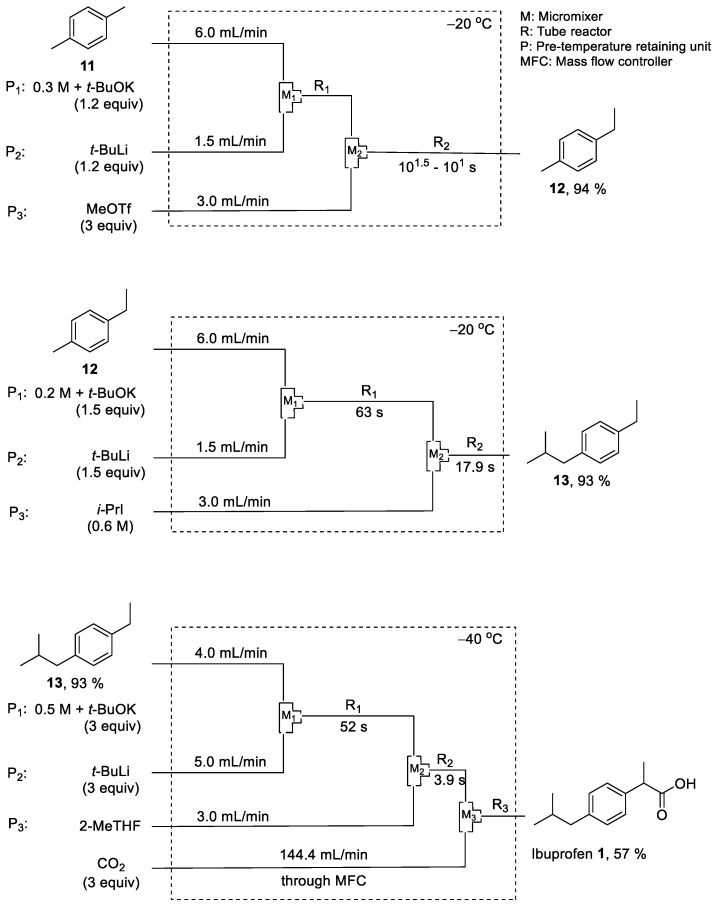
Continuous-flow synthesis of ibuprofen using C-H metalation. THF; tetrahydrofuran. Tf; trifluoromethansulfonyl.

Another approach employing continuous-flow synthesis, reported in 2019, is shown in Scheme 3 [25]. This synthesis involves direct alkylation of the benzylic anion via ‘superbase’-mediated deprotonation. Starting from *p*-xylene, in situ generated ‘superbase’ from KO*t*Bu and *t*BuLi afforded the desired benzylic anion to meet with adequate electrophiles sequentially. After 10 min, the process, which employed the use of MeOTf, *i*PrI, and CO_2_, produced 2.3 g ibuprofen (50% in three steps). The readily available starting material and repeated usage of the mixed base increases the cost-effectiveness of this approach. However, the strong basicity of this procedure still requires improvement for industrial purposes.

**Scheme 4:**
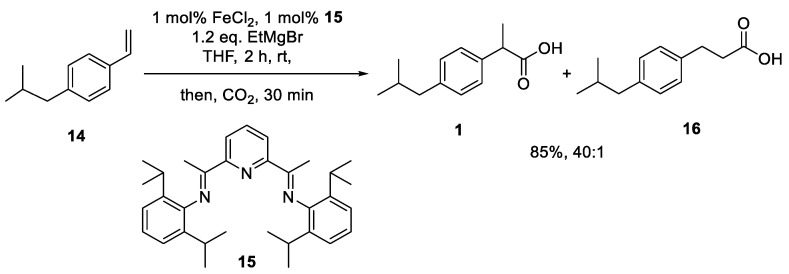
Iron-catalyzed hydrocarboxylation for ibuprofen synthesis.

The construction of a 2-arylpropionic acid skeleton inspired hydrocarboxylation, which is crucial for ibuprofen or naproxen synthesis [26]. Iron-catalyzed hydrocarboxylation, as reported by Thomas et al., is shown in Scheme 4 [27]. Although hydrocarboxylation of styrene was expected to introduce the ibuprofen skeleton, the regioselectivity hampered it [28]. However, the highly selective addition of CO_2_ was possible by employing an iron catalyst and pyridine ligand **15**. The following mechanistic study showed that the transmetallation and hydrometallation of iron and styrene moieties are important for regioselective addition.

**Scheme 5:**
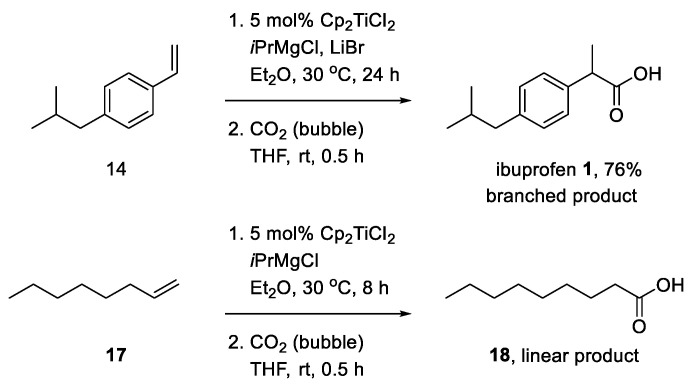
Titanium-mediated hydrocarboxylation for ibuprofen synthesis. Cp; cyclopentadienyl.

Hydrocarboxylation of styrene using Cp_2_TiCl_2_ catalyst, reported by the Xi group in 2016, is shown in Scheme 5 [29]. For this approach, the regioselectivity of the styrene moiety was screened using various Grignard reagents and additives. When this reaction was applied to alkyl-substituted alkenes, reversed regioselectivity was observed to have resulted in a linear product, nonanoic acid **18**.

**Scheme 6:**
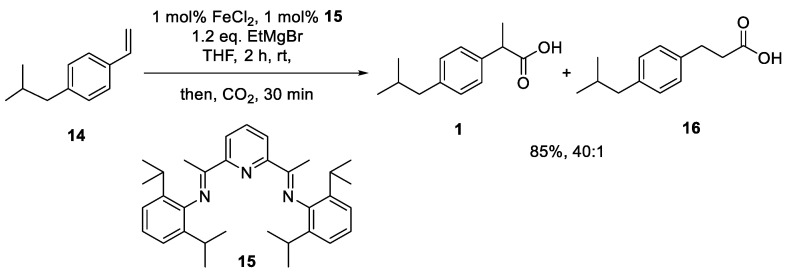
Iron-catalyzed regioselective carboxylation for ibuprofen synthesis. Ts; p-toluenesulfonyl.

A similar approach reported by Wang and Li’s groups in 2018 is shown in Scheme 6. They attempted to insert carbon monoxide (CO) into styrene **14** [30]. For a high regioselectivity of styrene, many cocatalysts, including metals or acids, were tested. When iron chloride (FeCl_3_) was used as the cocatalyst, a highly selective addition of CO was performed with approximately 90% yield. Arylethanol **19** was also used in the reaction system. Interestingly, the regioselectivity was fully reversed when the iron cocatalyst was changed from iron bromide to iron triflate. Although mechanistic studies are still required, versatile application of this strategy could be used as a valuable lead compound in drug discovery.

**Scheme 7:**
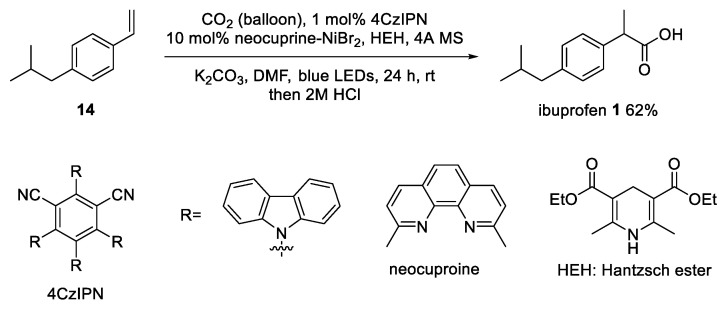
Visible light combining regioselective carboxylation for ibuprofen synthesis. 4CzIPN; 2,4,5,6-Tetrakis(9H-carbazol-9-yl) isophthalonitrile, *N*,*N*-dimethylformamide.

Regioselective hydrocarboxylation was also performed using a nickel catalyst in the presence of visible light, such as a blue LED [31], and is shown in Scheme 7. Hantzsch ester, molecular sieves, and nickel catalyst were added with the aid of visible light. Conversely, König group used a neocuproine ligand to amplify the regioselectivity of this reaction. When other phosphine ligands such as 1,4-bis(diphenylphosphino)butane (dppb) were used instead of neocuproine, a linear addition product was obtained exclusively. Additionally, they also performed mechanistic studies to elucidate the in situ generation of nickel-hydride complexes that are responsible for irreversible addition to styrene. Although this reaction system uses various ligands for ibuprofen synthesis, visible-light-assisted room-temperature reactions may be valuable for environmentally friendly industrial synthesis [32,33,34].

**Scheme 8:**
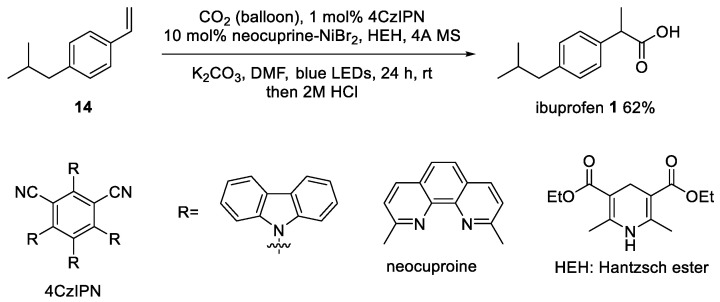
Reductant-free coupling for ibuprofen or naproxen synthesis.

A similar result was published in 2018; however, its approach differed in that no external reductant, such as Hantzsch ester, was necessary [35]. The replacement of benzylic quaternary ammonium salt by carboxylic acid functionality through an iridium-catalyzed carbonylation process is shown in Scheme 8 [36,37,38]. Upon using a blue LED, a smooth conversion to the desired product was observed. Notably, the reaction was completed without the need for an additional reducing agent, similar to the previous conversion. It was designed such that the released amine-leaving group might act as an electron donor. Additionally, naproxen **2** was successfully obtained by employing this reaction condition.

**Scheme 9:**
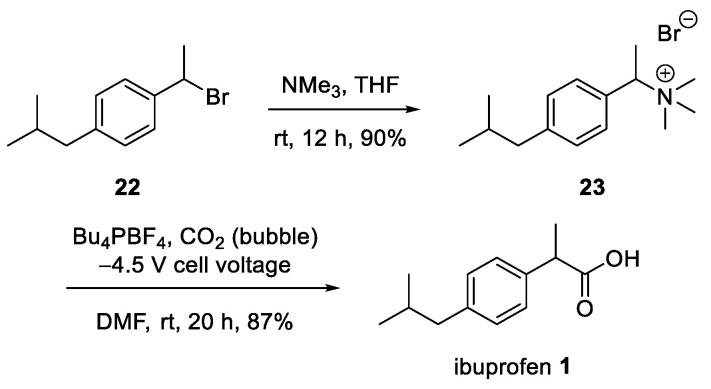
Electrochemical carboxylation for ibuprofen synthesis.

The carboxylation of quaternary ammonium salt, achieved by a direct electrochemical reaction with CO_2_, is shown in Scheme 9 [39]. Although this conversion shows a similar reaction pathway, it features electrochemical coupling of quaternary ammonium bromide **23** from benzylic bromide **22** [40] with CO_2_ without further metal catalysts, complex ligands, or external reducing agents. As the voltage apparatus is renewable [41], this type of conversion could provide a solution for the green synthesis of NSAIDs [42].

**Scheme 10:**
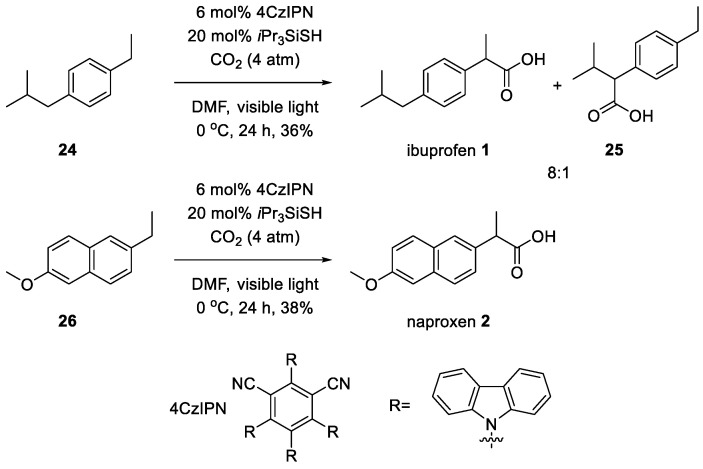
Photo-chemical carboxylation of alkyl benzene substrate for ibuprofen or naproxen synthesis.

A more direct CO_2_ addition from benzylic C-H activation through photo-activation was performed in 2019. After König et al. reported blue LED-mediated photocarboxylation (as shown in Scheme 7), they also attempted to add CO_2_ to the alkyl benzene substrate, which is readily available from commercial sources [43,44,45]. Scheme 10 shows the results of this endeavor [46]. Triisopropylsilyl thiol was used to facilitate hydrogen atom transfer for practical conversion of the sp^3^-hybridized C-H bond to the carbanion intermediate. The resulting benzylic radical was then reduced to a carbanion intermediate via electron transfer from 4CzIPN. Finally, the resultant benzylic anion intermediate reacted with CO_2_ to produce the desired 2-arylpropionic acid skeleton. Visible light was necessary to complete the reaction [47].

However, the relatively low chemical yield hampers further application, along with unwanted isomers from ibuprofen synthesis, which is a problem that has not yet been solved. Nevertheless, this conversion method still presents a major opportunity because it can be achieved without a metal catalyst or bulky ligand under convenient reaction conditions. In addition, the naproxen skeleton can be accessed selectively using this reaction as it has only one benzylic carbon.

**Scheme 11:**
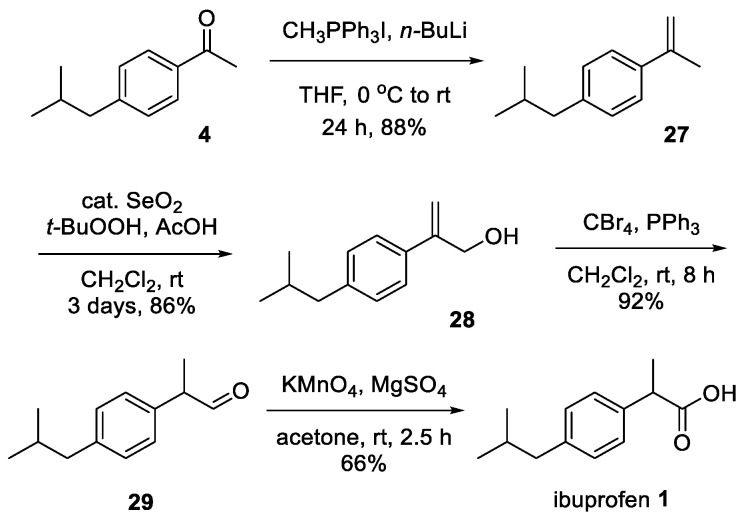
CBr_4_/PPh_3_-mediated isomeric oxidation protocol for ibuprofen synthesis.

Another synthetic protocol for ibuprofen synthesis is summarized in Scheme 11 [48]. It was attempted to substitute allylic alcohol **28** by the allylic oxidation of terminal methylene **27** using a simple Appel reaction [49]. However, they found that, unexpectedly, isomeric aldehyde **29** was obtained as the major product. After the mechanistic study and optimization of the reaction conditions, aldehyde **29** was produced with 92% yield, suggesting that uneventful oxidation of the aldehyde afforded ibuprofen in good yield. Although this reaction protocol requires more steps than the conventional BHC protocol, the scientific importance of various synthetic routes still exists.

**Scheme 12:**
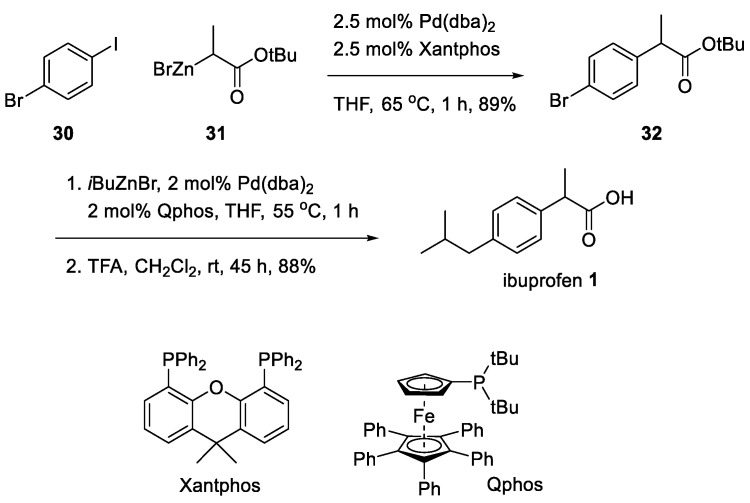
Reformatsky–Negishi approach for ibuprofen synthesis. Dba; dibenzylideneacetone, TFA; trifluoroacetic acid.

A chemoselective aromatic coupling approach has also been studied and is shown in Scheme 12 [50]. In 2014, Zhang group published sequential coupling with an aromatic halide with zinc enolate, followed by an alkyl zinc reagent. Although this procedure requires complex ligands and metals, the required reaction sequence is simple. Moreover, the simplicity of the starting material is also impressive. However, its iterative use of metals may hamper further development.

**Scheme 13:**
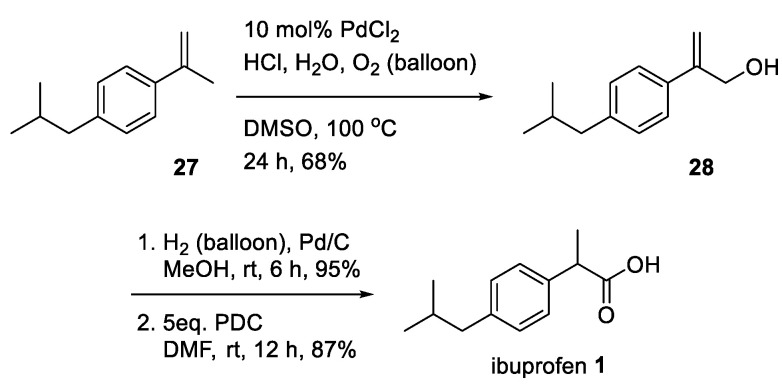
Aerobic oxidation approach for ibuprofen synthesis. DMSO; dimethylsulfoxide, PDC; pyridinium dichlorochromate.

A Pd-catalyzed allylic oxidation procedure was also developed and is shown in Scheme 13 [51]. Although this conversion can be performed using SeO_2_/*t*-BuOOH as described earlier, Jiang group used oxygen gas and catalytic PdCl_2_. The resulting allylic alcohol **28** could be transformed into ibuprofen by employing a reduction/oxidation sequence. A novel synthetic route for ibuprofen is thus possible.

**Scheme 14:**
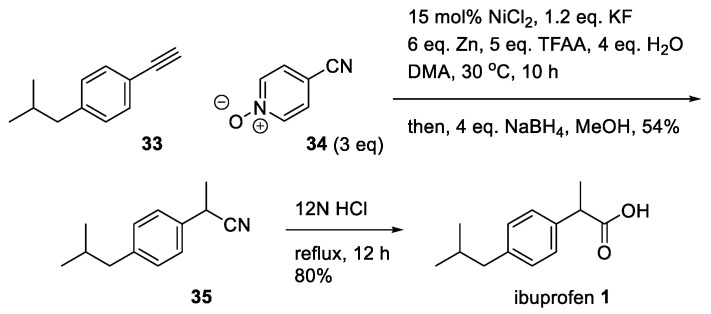
Alkyne-cyanation approach for ibuprofen synthesis. TFAA; trifluoroacetic anhydride, DMA; *N*,*N*-dimethylacetamide.

Another impressive strategy was developed by adding cyanide to aromatic alkynes [52] as shown in Scheme 14. For Markovnikov’s addition of cyanide, 4-cyanopyridine *N*-oxide **34** was adapted for cyanide supply. After the addition of cyanide to afford terminal methylene, it underwent one-pot reduction with NaBH_4_. Therefore, it was possible to produce substituted benzyl cyanide **35** using this strategy, which could be transformed into ibuprofen via simple acidic hydrolysis. However, this protocol employs excess reagents, including zinc. In this regard, further research is still required. However, its unique synthetic approach and moderate chemical yield are highly valuable.

**Scheme 15:**
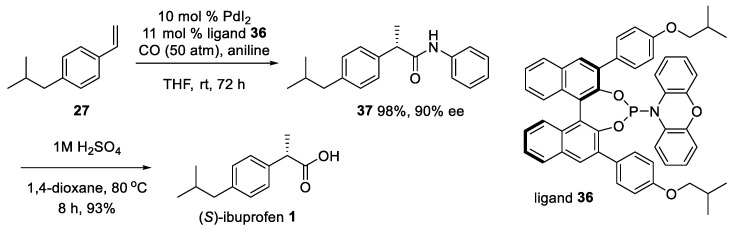
Asymmetric synthesis of (*S*)-ibuprofen.

Although ibuprofen has been commercialized as a racemic mixture, enantiomerically pure (*S*)-ibuprofen has better efficacy and potency. In line with this merit, synthesis of (*S*)-ibuprofen has been studied extensively. Scheme 15 is a good example of this endeavor. Incorporation of CO and aniline into the styrene **27** was accomplished enantiomerically pure manner [53]. It features asymmetric Markovnikov-type hydroaminocarbonylation of styrene with catalysis of Pd and phosphoramidite ligand **36**. Although this type of addition reaction has been reported, it is impressive that this reaction can be achieved at room temperature, with high regioselectivity (branched:linear = 99:1) and stereoselectivity (90% ee).

## 4. Classical Synthesis of Naproxen

**Scheme 16:**
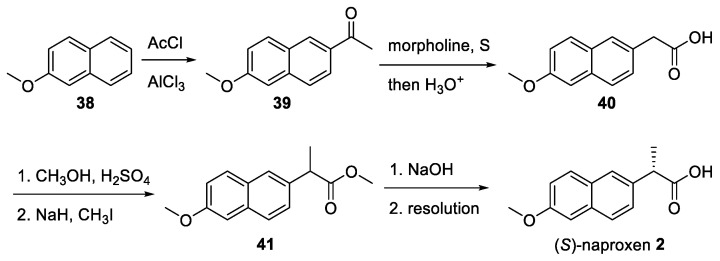
Classical synthesis of (*S*)-naproxen.

The classical synthesis of naproxen is shown in Scheme 16. Whilst ibuprofen is utilized as a racemic mixture, the utilization of naproxen is optically active. Therefore, chiral resolution [54] or asymmetric synthesis [55,56,57,58] is necessary in early development. When Syntex introduced naproxen in 1976, a synthetic procedure was adopted based on the traditional Friedel–Crafts alkylation [59] and Willgerodt–Kindler rearrangement [60] to afford arylacetic acid **40**. Esterification and simple methylation directly produced racemic arylpropionic ester **41**. Finally, saponification and chiral resolution using cinchonidine resulted in (*S*)-naproxen in an enantiomerically pure form [61].

## 5. Recent Synthetic Advances in Naproxen

**Scheme 17:**
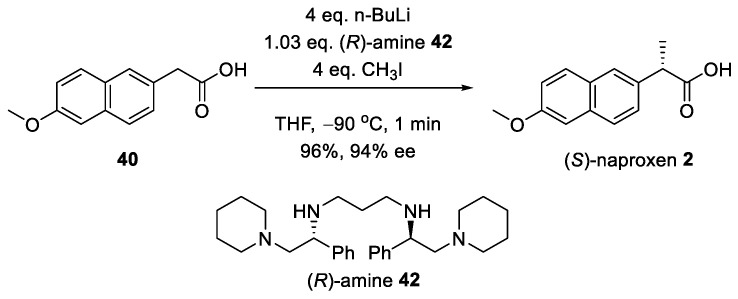
Chiral enolate strategy for (*S*)-naproxen synthesis.

In studies of naproxen synthesis, the asymmetric induction of chiral centers and construction of an arylpropionic acid skeleton are important. Zakarian et al. reported direct methylation of (*S*)-naproxen using a chiral enolate, as shown in Scheme 17. In contrast to conventional chiral alkylation requiring chiral auxiliary attachment and detachment [62,63], this chiral enolate (reagent) gives the desired product directly in a highly stereoselective manner. Although the author reported that the recovery of chiral amines was possible and readily available [64], excess use of base and electrophile needs to be improved for further application and industrial-scale synthesis.

**Scheme 18:**
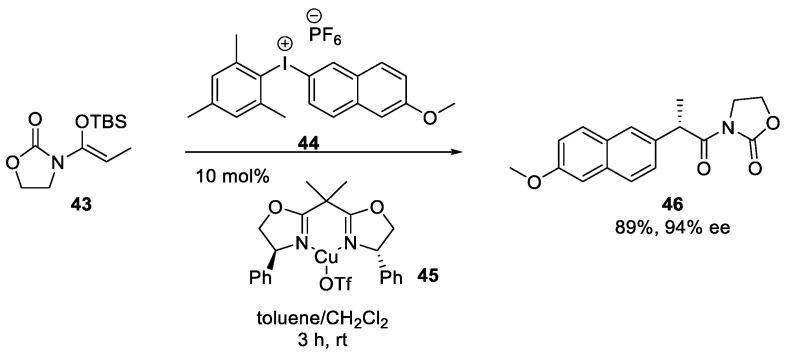
Asymmetric alkylation strategy using iodonium salt for (*S*)-naproxen synthesis. TBS; *t*-butyldimethylsilyl.

Diaryliodonium salt was also used for the synthesis of (*S*)-naproxen, as shown in Scheme 18 [65]. For enantioselective α-arylation of the carbonyl group [66,67,68], silyl ketene *N*,*O*-aminal **43** was chosen as the nucleophile, while reactive aryliodonium salt **44** was used as the electrophile [69,70]. When the nucleophile and electrophile were mixed with a chiral copper catalyst, the desired (*S*)-arylpropionic acid skeleton was enantiomerically pure. While this reaction can be performed in a one pot reaction efficiently, the resource requirements of diaryliodonium electrophile **44** [71,72] and non-atom economical nucleophile **43** still need further improvement.

**Scheme 19:**
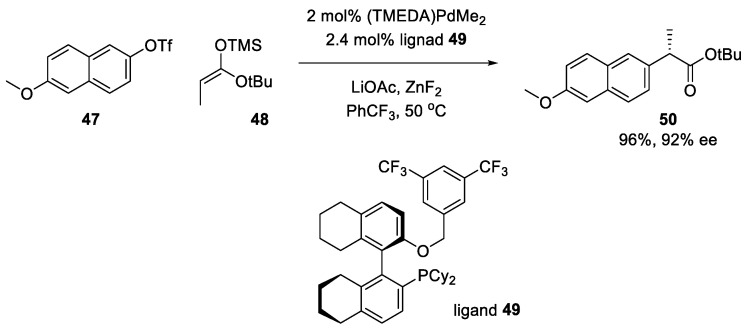
Asymmetric Pd-coupling strategy for (*S*)-naproxen synthesis. TMS; trimethylsilyl, TMEDA; tetramethylethylenediamine, Cy; cyclohexyl.

A similar approach was studied using Pd-mediated alkylation of silyl ketene acetal, as shown in Scheme 19 [73]. For this conversion, aryl triflate was used as the coupling reagent with a simple ketene acetal **48** in the presence of a Pd catalyst. More importantly, a chiral ligand **49** dominantly directs facial selectivity to produce the (*S*)-arylpropionic acid framework **50** [74]. This strategy also showed that other NSAIDs, such as (*S*)-fenoprofen, (*S*)-flurbiprofen, and (*S*)-ketoprofen, could be obtained in good chemical yield and stereoselectivity (>90% yield, >88% ee).

**Scheme 20:**
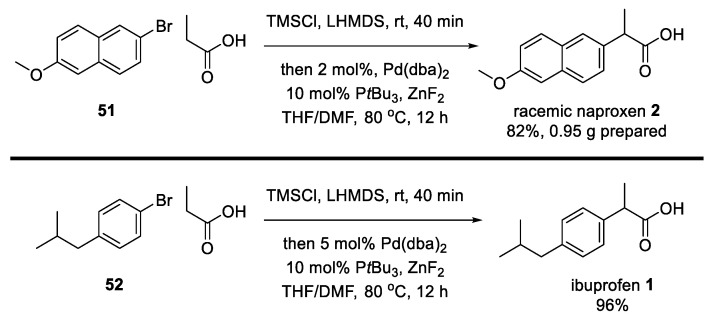
Pd-coupling for racemic naproxen or ibuprofen synthesis. LHMDS; lithium bis(trimethylsilyl)amide.

Pd-catalyzed coupling of aromatic halides with carboxylic acids has played an important role in organic synthesis [75,76,77]. Employing Pd-coupling technology in this regard, Hartwig group showed that aryl bromide **51** could react with propionic acid via a TMS-enolate intermediate [78] to obtain an arylpropionic acid skeleton with an excellent yield [79] (Scheme 20). This conversion does not require a highly complex ligand or reaction system. It was possible to synthesize racemic naproxen in gram scale or ibuprofen in over 95% yield using a simple procedure. Although this procedure still requires further advances in asymmetric synthesis, its ability to construct a carbon framework with an efficient methodology is noteworthy.

**Scheme 21:**
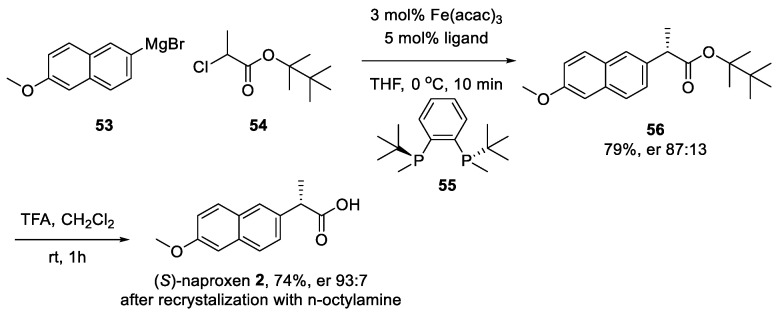
Iron-catalyzed chiral coupling to α-haloester strategy for (*S*)-naproxen synthesis. Acac; acetylacetonate.

α-Haloester was also utilized in the coupling reaction to afford (*S*)-naproxen [80,81]. Nakamura et al. reported that an arylmagnesium reagent could react with α-chloroester **54** with the assistance of an iron catalyst [82,83,84] and chiral phosphine ligand **55** to afford enantiomerically enriched ester **56**. For increased selectivity, an uncommon ester was used, as it could be converted to (*S*)-naproxen under acidic conditions [85] (Scheme 21). A mechanistic investigation was also performed to elucidate the radical intermediate and divalent iron species important for the catalytic cycle. The paper also includes that this reaction can be applied to (*S*)-ibuprofen synthesis in 65% yield with over 99% enantiopurity.

**Scheme 22:**
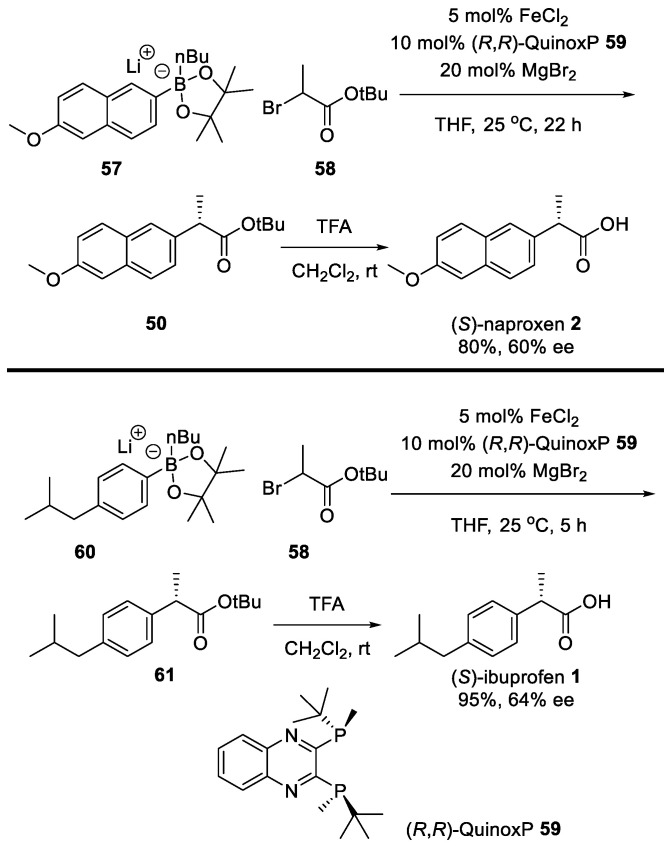
Iron-catalyzed Suzuki–Miyaura coupling for (*S*)-naproxen or (*S*)-ibuprofen synthesis.

Nakamura et al. further modified the iron-catalyzed coupling reaction to enantioselective Suzuki–Miyaura coupling with α-bromoester **58**, as shown in Scheme 22 [86]. Considering the efficiency of aryl borates for metal-catalyzed coupling, this type of chiral coupling can be utilized for practical synthesis. Chiral coupling followed by simple acidic hydrolysis assessed both enantiomerically enriched (*S*)-naproxen and (*S*)-ibuprofen. However, readily available chiral ligands and high selectivity are still required.

**Scheme 23:**
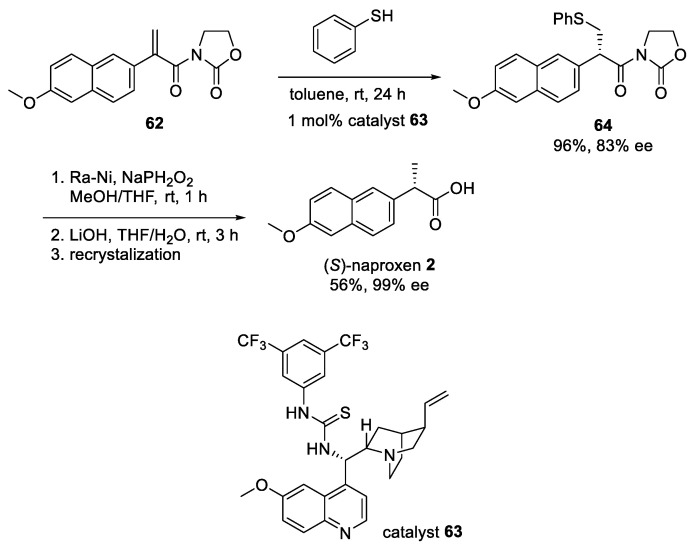
Asymmetric addition of thiol for (*S*)-naproxen synthesis. Ra-Ni; Raney-nickel.

The asymmetric addition of thiophenol was also attempted, as shown in Scheme 23 [87]. The α,β-unsaturated terminal alkene **62** was treated with thiophenol to produce a Michael adduct **64**. This conversion proceeded asymmetrically with the influence of cinchona-derived catalyst **63**. Raney-Ni and NaPH_2_O_2_ were treated to cleave the carbon-sulfur bond in the next step. Finally, hydrolysis and recrystallization afforded enantiomerically pure (*S*)-naproxen in good yields. Employing this strategy, (*S*)-ibuprofen could also be obtained with a similar yield and enantiomeric purity. Thus, the author proposed that thiourea as a chiral catalyst could interact with the dicarbonyl groups in the substrate via hydrogen bonding, serving as a chiral platform for the asymmetric Michael addition of thiophenol.

**Scheme 24:**
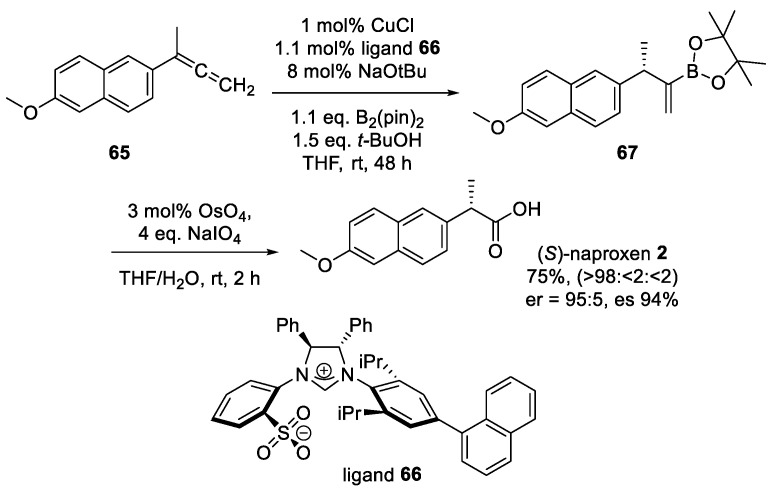
Hydroboration of allene strategy for (*S*)-naproxen synthesis. Pin; pinacol.

The synthesis of alkenyl boron via hydroboration of 1,1-disubstituted allenes was also pursued, as shown in Scheme 24 [88,89,90]. Hoveyda et al. reported that asymmetric hydroboration of allene **65** could be achieved by employing a copper catalyst, *N*-heterocyclic carbene (NHC) ligand **66**, and pinacolborane. When the reaction was performed at room temperature for 2 d, the desired alkenyl pinacolatoboron **67** could be produced with excellent enantio-and regioselectivity. Finally, the oxidation of alkenes using OsO_4_ followed by NaIO_4_ afforded (*S*)-naproxen directly. Mechanistic investigations and further applications are currently in progress [91].

**Scheme 25:**
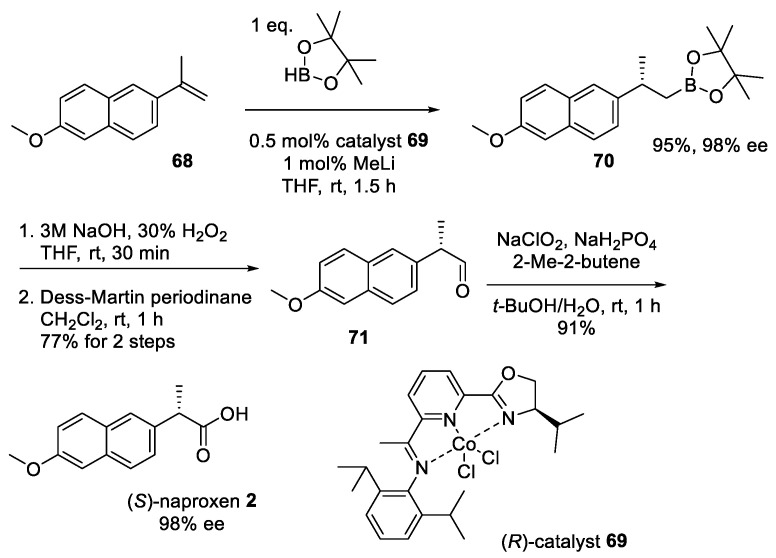
Hydroboration of terminal alkene strategy for (*S*)-naproxen synthesis.

Another asymmetric hydroboration route to (*S*)-naproxen was developed in 2014 [92]. The terminal alkene **68** was chosen for pivotal hydroboration to use a more versatile substrate. Remarkably, cobalt catalyst **69** was prepared for this conversion after an extensive screening of the catalyst and reaction conditions. After the desired pinacolatoboron **70** was obtained exclusively, it was oxidized to (*S*)-naproxen via a three-step sequence without any epimerization. The overall yield was 66% from the starting alkene **68**, as shown in Scheme 25. Hence, considering the easy preparation of chiral catalysts and their low loading, this reaction could be considered for the large-scale synthesis of other NSAIDs if cobalt can be treated in an environmentally friendly manner.

**Scheme 26:**
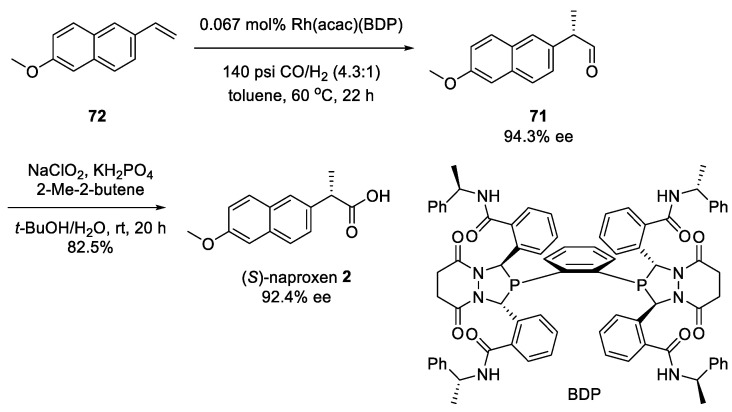
Asymmetric hydroformylation strategy for (*S*)-naproxen synthesis. BDP; (*R*,*R*,*R*)-Bisdiazaphos-SPE.

Hydroformylation in flow reactions has also been studied for industrial purposes [93,94,95]. Researchers at the University of Wisconsin-Madison and Eli Lilly Corporate Center published a paper on the asymmetric synthesis of (*S*)-naproxen using rhodium-catalyzed hydroformylation, as shown in Scheme 26 [96]. In this paper, gas and liquid reagents were mixed in a flow reactor. After a systematic survey of the reactor design and chiral ligands, (*S*)-naproxen was produced in two steps in over 80% yield and 92% enantiomeric excess from the simple vinyl substrate **72**. As this reaction is designed to work in a continuous-flow reactor system, its use for the industrial synthesis of (*S*)-naproxen is also possible [97].

**Scheme 27:**
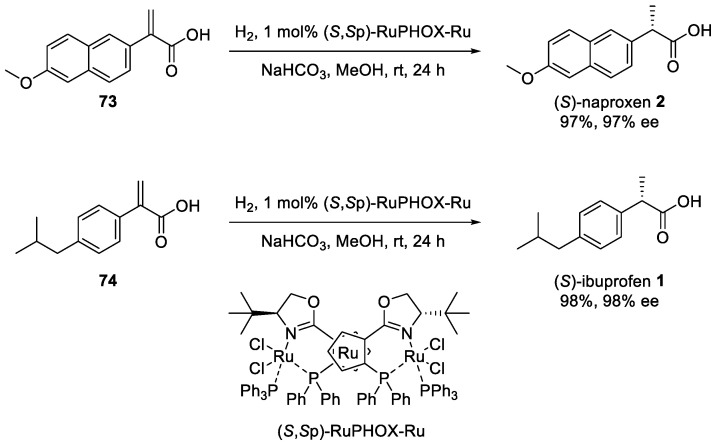
Asymmetric hydrogenation strategy for (*S*)-naproxen or (*S*)-ibuprofen synthesis.

Asymmetric hydrogenation is a valuable methodology for chiral medicine synthesis [98,99,100]. For NSAID synthesis, the chiral ferrocene-ruthenium complex was reported to be promising, as shown in Scheme 27 in 2016 [101]. This reduction process was developed for NSAID synthesis and other chiral propionic acid skeletons, such as artemisinin. In some examples, catalyst loading could be reduced to 0.02 mol%; therefore, further development and wide applications are promising. Moreover, (*S*)-naproxen and (*S*)-ibuprofen were obtained in over 97% chemical yield and over 97% enantiomeric purity.

**Scheme 28:**
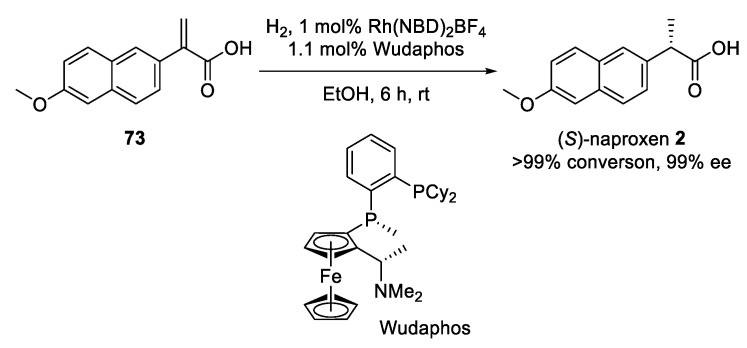
Rhodium-catalyzed asymmetric hydrogenation for (*S*)-naproxen synthesis.

Other chiral ligands for asymmetric hydrogenation have been reported simultaneously [102]. Pursuing high enantioselectivity and low catalyst loading, a new class of biphosphorus ligand with a ferrocene moiety was developed and screened. Wudaphos was chosen after a survey of the chiral ligands with a rhodium catalyst for asymmetric reduction (Scheme 28) [103]. Mechanistically, this ligand was designed to interact via hydrogen bonding of the ammonium ion of the ligand and carboxylate ion of the substrate. It is noteworthy that the turnover number (TON) reaches 20,000. Moreover, its high efficiency has received increased industrial attention for chiral NSAIDs or other active pharmaceutical ingredients (APIs).

**Scheme 29:**
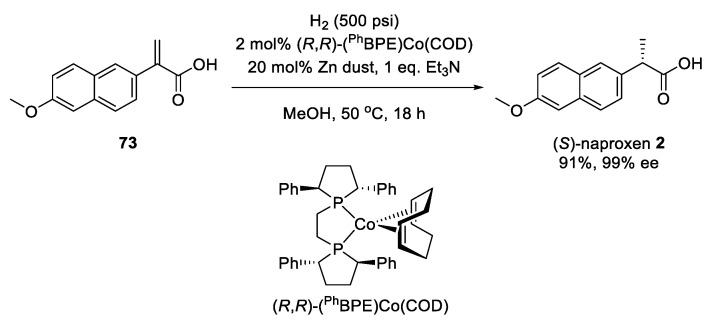
Cobalt-catalyzed asymmetric hydrogenation for (*S*)-naproxen synthesis.

Cobalt-catalyzed asymmetric hydrogenation was also studied, as shown in Scheme 29. During his continuous research on cobalt-catalyzed asymmetric reduction [104], Chirik et al. reported that the addition of hydrogen could be enantioselective via cobalt-mediated catalysis [105]. A mechanistic study was also conducted. Carboxylic acid was added to the cobalt complex as a ligand, and the face was selectively reduced. It was validated by X-ray crystallography to elucidate the intermediate structure of the catalyst. This highly effective methodology is compatible with various substrate functionalities.

**Scheme 30:**
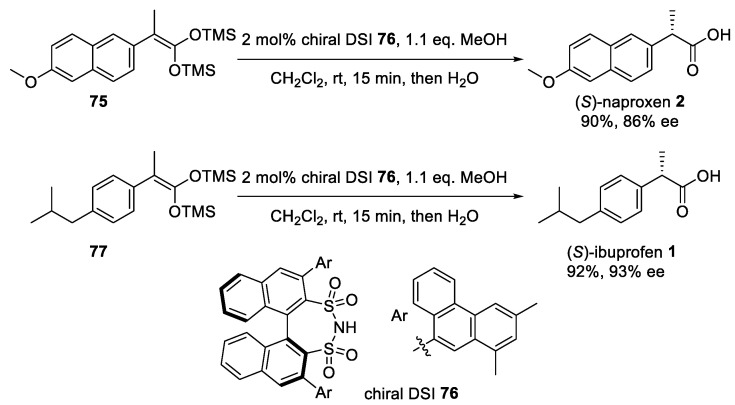
Asymmetric protonation strategy for enantiomerically enriched (*S*)-naproxen or (*S*)-ibuprofen synthesis. DSI; disulfonimide.

For the asymmetric synthesis of NSAIDs, a different approach was realized by List et al. in 2019. As shown in Scheme 30 [106], chiral protonation of bis-TMS ketene acetal **75** was attempted to afford a new stereogenic center [107,108,109,110]. The face-selective protonation could be accomplished in a high ratio by using chiral disulfonimide **76** proton donors. Similarly, isobutyl analog **77** was successfully transformed into (*S*)-ibuprofen. However, this deracemization route has the drawback that it still requires complete construction of a final racemic compound. In addition, they must compete with traditional and well-established chiral resolution routes. Nevertheless, the low catalyst loading and simple procedure of this asymmetric protonation process still indicates its potential for further application and development.

**Scheme 31:**
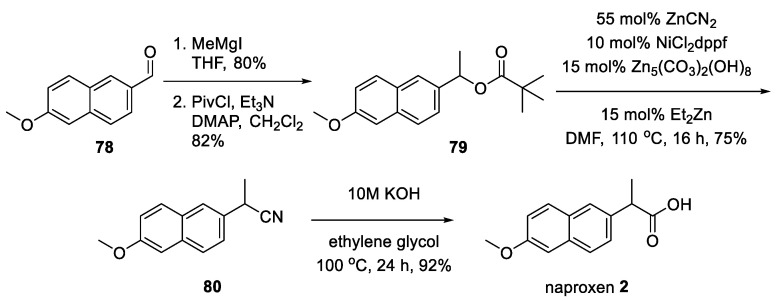
Ni-catalyzed cyanation strategy for naproxen synthesis. Piv; pivaloyl, DMAP; 4-dimethylaminopyridine, dppf; 1,1′- bis(diphenylphosphino)ferrocene.

Another synthetic procedure for racemic naproxen was reported in 2018, as shown in Scheme 31 [111]. This procedure features nickel-catalyzed cyanation of benzyl pivalate **79** through methylation and esterification of the resulting secondary alcohol starting from an aromatic aldehyde **78**. Zinc cyanide was used as a 0.55 equivalent for low cyanide loading. It is also interesting that the use of zinc ligands suppresses the competing elimination process. Therefore, racemic naproxen was efficiently produced with this four-step synthesis.

**Scheme 32:**
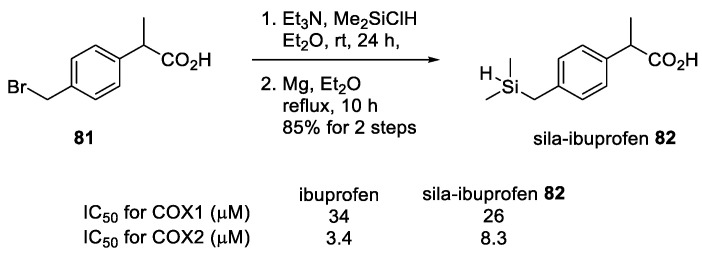
Introduction of silicon in ibuprofen structure.

Based on the synthetic advance described above, more promising derivatives of ibuprofen and naproxen are still studied. Scheme 32 presents an interesting showcase. Grabowsky and Beckmann’s group reported substitution of silicon instead of carbon at alkyl side chain in ibuprofen structure. This simple silicon-substituted ibuprofen **82** showed improved physical property such as solubility in body [112]. Although selectivity on COX1/COX2 is still demanding, this simple and direct change of structure can be another solution for further development.

## 6. Conclusions

Since the discovery of aspirin on 10 August 1897, anti-inflammatory medicines have attracted tremendous attention from pharmacologists and medicinal chemists. After the elucidation and differentiation of COX into COX1 and COX2, mechanistic studies were deeply investigated. However, a breakthrough in chemical synthesis was essential for more selective and potent medicines, as the Kolbe–Schmidt reaction occurred in 1859. The reaction suggested that salicylic acid is not supplied from salicin, a natural source, but from a chemical compound, phenol, NaOH, and CO_2_. It is more impressive that the structural modification of salicylic acid was extensively pursued using the confirmed structure of salicylic acid and its easy availability. Thus, this synthetic breakthrough paved the way for the discovery of aspirin.

However, there is still a need for further innovation in NSAID drug development, as the Kolbe–Schmidt reaction did. Gratifyingly, enormous chemical studies on asymmetric synthesis, large-scale preparation, and new methodology development are still ongoing. Based on the achievement, NSAIDs such as ibuprofen or naproxen could be purchased without a price issue nowadays. However, novel demonstrations of a newly developed methodology for ibuprofen or naproxen skeleton are still a good showcase of organic/medicinal chemistry research.

In addition, medicinal chemists have tried to find advanced NSAIDs with regard to safety and efficacy. To find selective COX2 inhibitor without undesired side effects, tremendous research is underway. This endeavor includes a simple exchange of atom as well as a modification of mother skeleton in NSAIDs. Thus, it is anticipated that there will be a promising breakthrough in the near future.

## Data Availability

Not applicable.

## Sample Availability

Samples of the compounds are available from the authors.
